# Teach plants to fish based on CRISPR‐Cas system self‐evolution

**DOI:** 10.1111/pbi.70066

**Published:** 2025-03-21

**Authors:** Xuhui Ma, Liqing Miao, Xiaoqing Liu

**Affiliations:** ^1^ Isotope Institute Co., Ltd, Henan Academy of Sciences/Henan Key Laboratory of Nuclear Agricultural Sciences Zhengzhou Henan China; ^2^ Biotechnology Research Institute Chinese Academy of Agricultural Sciences Beijing China

**Keywords:** CRISPR‐Cas system, plant immunity, self‐evolution

The CRISPR‐Cas system serves as an adaptive immune system developed by bacteria and archaea over a lengthy period of evolution to defend against phage and foreign nucleic acid intrusion. However, the components of the CRISPR‐Cas system, including ‘clustered, regularly interspaced short palindromic repeats (CRISPR)’ and CRISPR‐associated genes (Cas), were first identified in 1987. The type II CRISPR‐Cas system, originating from *Streptococcus thermophilus*, was experimentally demonstrated to operate as an acquired immune system in 2007 (Barrangou *et al*., 2007). In 2011, Emmanuelle Charpentier and Jennifer A. Doudna collaborated on a groundbreaking experiment, confirming that CRISPR‐Cas9 can cleave DNA in vitro. Subsequently, George Church's lab and Feng Zhang's group achieved CRISPR genome editing in mammalian cells in 2013 (Cong *et al*., 2013), while three other research groups successfully employed CRISPR‐Cas9 to modify plant DNA in the same year (Shan *et al*., 2013). Since then, CRISPR‐Cas‐based genome editing technologies, such as CRISPR‐Cas9, CRISPR‐Cas12a, base editing, prime editing and RNA editing (Wang *et al*., 2020), have been extensively investigated and are widely utilized as precise tools for nucleic acid manipulation in various organisms.

CRISPR‐Cas‐based genome editing technologies offer various applications, including imparting virus resistance to plants (Ji *et al*., 2015; Yicheng *et al*., 2020). However, current applications only tap into a fraction of bacteria's natural defence system. The system's uptake, storage, and transcription of exogenous nucleic acids demonstrate even more intricate and intelligent functions that remain underdeveloped and underutilized. Consequently, current applications can only edit one or a few target sites in the recipient organism, leading to resistance against only one or several viruses. An ancient Chinese proverb known to all educators in China states: ‘Give a man a fish, and you feed him for a day; teach a man to fish and you feed him for a lifetime.’ Therefore, we propose that in the realm of utilizing genome editing technology to combat viral diseases, why not teach higher plants the ability to ‘fish’? If we can fully transplant bacteria's ability to immunize against viruses into plants, they may be able to uptake and store invading viral nucleic acid fragments in their genomes to form CRISPR regions, similar to bacteria. This could potentially equip plants with the capability to efficiently defend against multiple viruses. Moreover, this ability can be pre‐trained by cultivating materials with high antiviral resistance and then backcrossing them to generate varieties for agricultural production. Consequently, the antiviral spectrum can be broadened, and subsequent production can enhance broad‐spectrum and highly efficient antiviral capabilities even further.

## Design of an intelligent plant immune system

In plants, tobacco or Arabidopsis can be utilized to create transgenic plants harbouring the novel plant immune system. As known, the CRISPR‐Cas loci‐derived immune system operates in three key phases: (I) adaptation, (II) expression and (III) interference. Therefore, all functional genes involved in the immune system need to be modified for plant gene expression. In the first phase, Cas1 and Cas2 are the core genes for prespacer acquisition, while Cas4 and Csn2 act as supplemental genes for the Cas1‐Cas2 complex in type I and type II spacer acquisition systems, respectively (Nunez *et al*., 2015). After capturing prespacers, they must be integrated into the CRISPR array through different mechanisms depending on the Cas system. For instance, in type II systems, correct integration of prespacers is facilitated by the leader‐anchoring sequence (LAS) located between the leader sequence and the first direct repeat (McGinn and Marraffini, 2019). In type I‐E systems, in addition to LAS, integration host factor (IHF) is also necessary. Therefore, we can select Cas1‐Cas2‐Csn2 for prespacer acquisition and LAS for integration in type II systems, or Cas1‐Cas2‐Cas4, LAS‐IHF in type I systems. In the expression phase, all functional genes or the CRISPR array need to be expressed constitutively since most CRISPR‐Cas loci are constitutively expressed and are prepared to target incoming invaders immediately after their genome injection into bacteria. Hence, Cas1, Cas2 and other genes used for capturing can be optimized for plant codon preference and driven by constitutive expression promoters like the CaMV 35S promoter. The leader + simple CRISPR region (one or several DR + spacer repeats) can be driven by the plant U6 promoter responsible for small RNA transcription, as the leader may not function as a promoter in plant expression systems as it does in bacterial cells. To enhance the transcription efficiency of spacers located far from the U6 promoter due to the CRISPR region's length, multiple leaders and simple CRISPR regions can be established to accept spacers simultaneously. The final interference phase is relatively straightforward, requiring only the expression of effector proteins such as Cas9. If Cas9 is selected, indicating targeting of DNA viruses, a nuclear localization signal peptide (NLS) needs to be fused with the Cas9 coding region to guide Cas9 into the plant cell nucleus for functionality.

**Figure 1:**
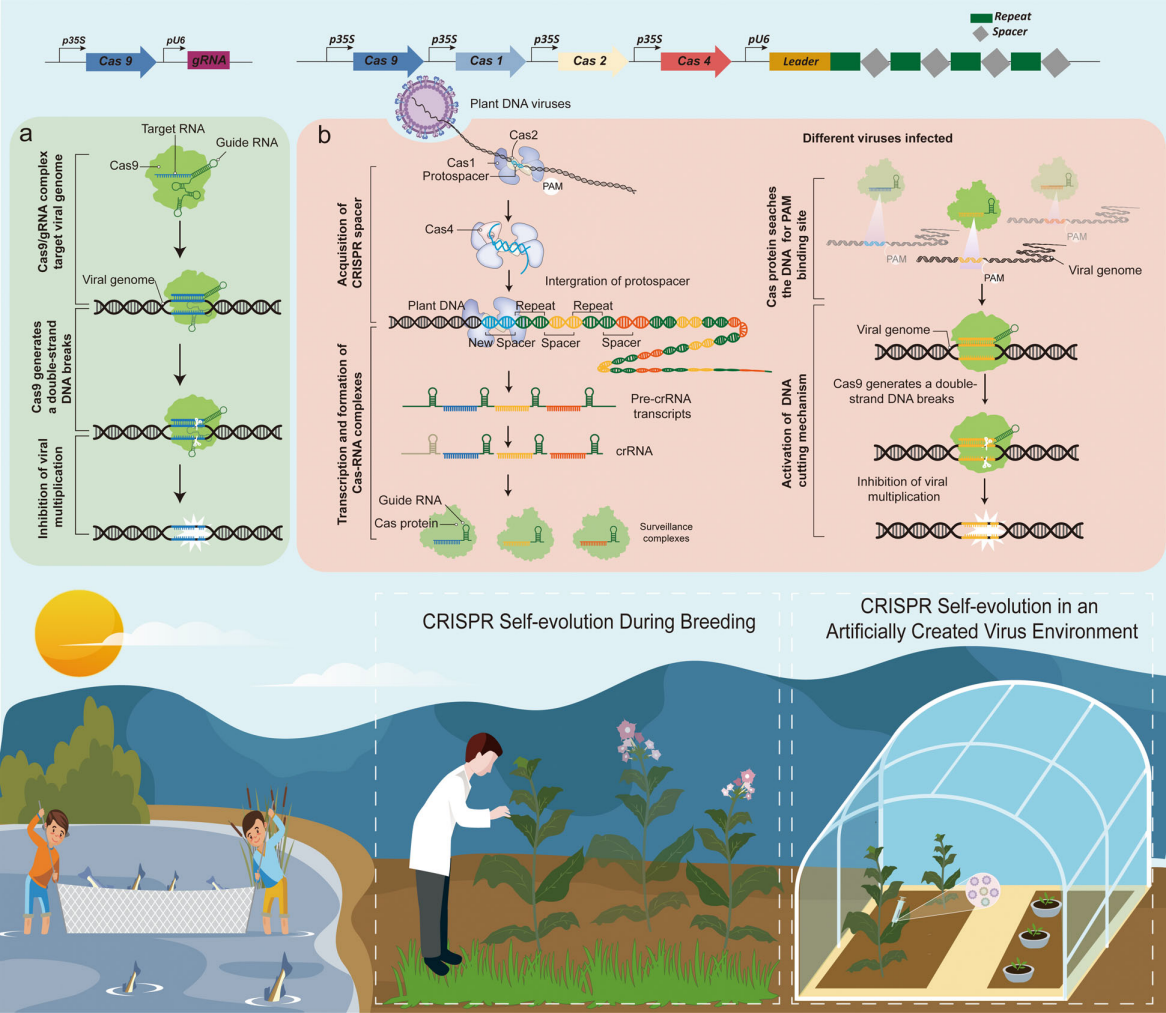
Conceptual diagram of intelligent plant immunity. (a) Classical CRISPR/Cas strategies targeting viral genomic DNA in plant cells. (b) New CRISPR/Cas strategies targeting viral genomic DNA in plants. Adaptive immune memory mechanism: During viral infection, Cas1‐Cas2 complexes integrate viral DNA fragments (spacers) into the plant’s CRISPR array, forming a pathogen‐specific memory. The array is transcribed and processed into mature crRNAs, which assemble with Cas effectors into surveillance complexes. Upon reinfection, these complexes recognize complementary viral sequences, guiding Cas nucleases to cleave invading DNA. This dual‐action mechanism blocks immediate viral replication and establishes long‐term immunity against future attacks.

When targeting RNA viruses, Cas13 would be selected as the effector protein. However, this system introduces significant complexity due to the spatial conflict between viral replication and CRISPR functionality: RNA viruses replicate in the cytoplasm, whereas spacer storage and CRISPR array transcription must occur in the nucleus. Therefore, all effector proteins involved in prespacer capture and the final editing process must be fused with both NLS and nuclear export signal peptide (NES) for continuous nuclear‐cytoplasmic shuttling. Moreover, as the prespacers derived from RNA viruses are composed of RNA while the CRISPR array is DNA‐based, spacer acquisition necessitates RNA‐to‐DNA conversion. This process could be mediated by a reverse transcriptase‐Cas1 (RT‐Cas1) fusion protein, enabling CRISPR spacer acquisition from RNA. Specifically, RT‐Cas1 and Cas2 would function to: acquire RNA prespacers in the cytoplasm; (Barrangou *et al*., 2007) transport the reverse‐transcribed DNA fragments to the nucleus; and integrate them into the CRISPR array (Figure 1).

## Detection of prespacer capture ability

After generating transgenic plants, such as tobacco, containing the novel immune system, like CRISPR‐Cas9 loci, the initial step involves testing whether the transgenic tobacco possesses the ability to capture prespacers. Initially, the DNA virus BSCTV can infect transgenic tobacco, and molecular detection can be conducted on tobacco leaves at various time points post‐infection to ascertain whether new spacers are integrated downstream of the leader. However, this could be complex as each cell of the transgenic tobacco harbours an independent set of novel immune systems, leading to diverse situations regarding acquired spacers. While some cells may not acquire any spacers, others may acquire multiple spacers. Additionally, some cells may have the same number of spacers but different types. Considering this cellular diversity, the overall acquisition of spacers can be analysed across three dimensions: individual plants, tissues, and cells, to determine if there is a hotspot effect and positional effect of spacers. Secondly, two or more different DNA viruses can be employed for simultaneous infection to detect the prespacers of the CRISPR region originating from different DNA viruses. Thirdly, both DNA viruses and RNA viruses can be simultaneously used for infection to observe if the plant can acquire prespacers from both types of viruses. In such scenarios, it would be necessary to introduce CRISPR‐Cas9 loci and CRISPR‐Cas13 loci simultaneously into the transgenic tobacco, given that the plant genome size is large enough to accommodate these immune systems. It is critical to address the limitation of CRISPR‐Cas13 systems caused by their collateral cleavage activity, which may compromise their practical applicability. Although studies have demonstrated the establishment of CRISPR/Cas13a‐based immune systems conferring RNA virus resistance in both dicot and monocot plants through engineered spacer design, further advancements are required.

## Validation of antiviral ability

Upon confirming that the transgenic tobacco effectively acquires prespacers from invading viruses post‐infection, the question arises regarding whether the offspring of this transgenic tobacco will manifest an immune response and develop resistance to the same virus. Given that the CRISPR region follows a constitutive expression pattern, it can indeed transcribe long transcripts. While Cas12 can recognize the DR and process it into mature crRNA (Fonfara *et al*., 2016), Cas9 lacks this capability. Consequently, the pre‐crRNA of Cas9 must be continuously processed by an additional ribonuclease, RNase III. With mature crRNA and constitutively expressed Cas9 or Cas12 targeting the corresponding organelle, if this heterologous reconstituted immune system functions properly, it can demonstrate disease resistance.

Firstly, transgenic and wild‐type tobacco can be inoculated with a specific virus and their response to the infection monitored. Observe symptoms, disease progression, and survival rates between the two groups. Reduced symptoms and higher survival rates in transgenic tobacco indicate their antiviral ability. Secondly, a comparative analysis of viral accumulation levels in transgenic and wild‐type tobacco plants inoculated with the same virus will be conducted. Lower viral accumulation levels in the transgenic plants suggest a higher antiviral ability. Finally, expose both the transgenic and wild‐type tobacco to natural viral infections. Monitor disease incidence, severity, and overall performance in the field. Lower disease incidence or severity in the transgenic materials compared to the control materials indicates their antiviral ability.

## Artificial acceleration and self‐evolution of antiviral ability

To meet the demands of agricultural production, enhancing plant disease resistance is paramount. By subjecting transgenic materials to controlled environments and exposing them to various viruses, their disease resistance can be accelerated. This training process involves artificially infecting the plants with viruses and selecting those exhibiting stronger resistance traits for further breeding. Once these trained plants are integrated into agricultural production, they can continue to strengthen their disease resistance through natural selection, facilitated by a functional and effective CRISPR array. As these plants are exposed to a variety of viruses in the field, they naturally evolve and adapt to their environment. This self‐evolution process allows the plants to expand their CRISPR array further, enhancing their resistance to diseases over time. The combination of artificial acceleration of disease resistance training and subsequent self‐evolution plays a vital role in ensuring sustainable and resilient agricultural production. By continuously enhancing their disease resistance, plants can better withstand various pathogens, contributing to the overall health and productivity of agricultural systems. These strategies embody the proverb ‘Give a man a fish, and you feed him for a day; teach a man to fish, and you feed him for a lifetime’.

## Conflict of interest

The authors declare no conflict of interest.
